# Treatment of mesial temporal lobe epilepsy using MRgFUS: laboratory feasibility study

**DOI:** 10.1186/2050-5736-3-S1-O28

**Published:** 2015-06-30

**Authors:** Stephen Monteith, John Snell, Matt Eames, David Newell, Ryder Gwinn

**Affiliations:** 1 Swedish Neuroscience Institute, Seattle, WA, United States; 2 Focused Ultrasound Foundation, Charlottesville, VA, United States

## Background/introduction

Temporal lobe epilepsy remains a significant cause of disabling morbidity. Patients that are refractory to medical treatment are often considered for invasive surgery to remove the epileptogenic focus originating from the structures of the medial temporal lobe. A range of surgical options now exist with varying degrees of efficacy. The goal of any surgical intervention is to minimize disruption of adjacent normal cortical tissue while removing the offending structures. MRgFUS provides the opportunity to perform this task in an elegant fashion. We tested the feasibility of this approach in a cadaveric model.

## Methods

Cadaveric skulls were filled with custom fitted thermo reactive gels and thermocouples placed along anatomical areas of interest along the skull base to assess temperature rises during the sonication process using the InSightec ExAblate Neuro System (650 Khz). The volume of temporal lobe structures typically removed by standard surgical excision was mapped on 3D volumetric MRI scans and overlaid onto the thermal gels to provide an appropriate target for ablation. Temperature maps in the treatment volume were created for various sonication parameters and temperature effects at key skull base structures were monitored with thermocouples.

## Results and conclusions

By adjusting sonication parameters it was possible to create a lesion of therapeutic significance in the required temporal lobe treatment volume using thermoreactive gels in cadaveric skulls. Temperature rises seen at the periphery of the target volume did decrease compared to more medial structures. Treatment of temporal lobe epilepsy with MRgFUS appears feasible based on this laboratory model. Thoughtful planning using a disconnection strategy or highly focused sonications on key anatomical epileptogenic foci may be required due to the large volume required to ablate an equivalent volume for an anatomic temporal lobectomy. Blocking of distant FUS beams may be required to circumvent temperature rises in the anterolateral temporal fossa.

